# New Insights in Mechanisms and Therapeutics for Short- and Long-Term Impacts of Hepatic Ischemia Reperfusion Injury Post Liver Transplantation

**DOI:** 10.3390/ijms22158210

**Published:** 2021-07-30

**Authors:** Hui Liu, Kwan Man

**Affiliations:** Department of Surgery, HKU-SZH & Faculty of Medicine, The University of Hong Kong, Hong Kong, China; u3002399@connect.hku.hk

**Keywords:** liver ischemia reperfusion injury, inflammation, immune responses, metabolism, therapeutic strategies

## Abstract

Liver transplantation has been identified as the most effective treatment for patients with end-stage liver diseases. However, hepatic ischemia reperfusion injury (IRI) is associated with poor graft function and poses a risk of adverse clinical outcomes post transplantation. Cell death, including apoptosis, necrosis, ferroptosis and pyroptosis, is induced during the acute phase of liver IRI. The release of danger-associated molecular patterns (DAPMs) and mitochondrial dysfunction resulting from the disturbance of metabolic homeostasis initiates graft inflammation. The inflammation in the short term exacerbates hepatic damage, leading to graft dysfunction and a higher incidence of acute rejection. The subsequent changes in the graft immune environment due to hepatic IRI may result in chronic rejection, cancer recurrence and fibrogenesis in the long term. In this review, we mainly focus on new mechanisms of inflammation initiated by immune activation related to metabolic alteration in the short term during liver IRI. The latest mechanisms of cancer recurrence and fibrogenesis due to the long-term impact of inflammation in hepatic IRI is also discussed. Furthermore, the development of therapeutic strategies, including ischemia preconditioning, pharmacological inhibitors and machine perfusion, for both attenuating acute inflammatory injury and preventing late-phase disease recurrence, will be summarized in the context of clinical, translational and basic research.

## 1. Introduction

End-stage liver disease is a growing cause of mortality worldwide [1]. Liver transplantation has been established as the best curative treatment for patients with a wide range of acute and chronic liver diseases. However, hepatic ischemia reperfusion injury (IRI) represents a major risk factor which worsens the survival of liver transplant patients and exacerbates the shortage of donors [2]. Liver IRI initiates inflammation post transplantation. The short-term effects of inflammation lead to liver graft dysfunction or nonfunction and a higher incidence of acute rejection. The long-term impact of inflammation causes chronic rejection, impairment of regeneration, cancer recurrence and fibrosis development [3].

The pathological mechanisms of inflammation induced by liver IRI involve various processes such as cell death programs, metabolic alteration and immune activation. The cell death programs include apoptosis, necrosis, ferroptosis and pyroptosis, which release the danger-associated molecular patterns (DAMPs). These processes change the mitochondrial metabolism of liver graft at the acute phase and subsequently initiate the immune responses [3]. The innate immune responses play a major role in hepatic IRI. Moreover, a robust adaptive immune activation dependent on CD4^+^ T cells is also elicited [4]. Therefore, both innate and adaptive immune responses participated in the process [5]. The immune cells involved in hepatic IRI not only induce the acute-phase inflammation but also change the microenvironment of the liver graft, which subsequently leads to the long-term outcome.

Thus, understanding the mechanisms of inflammation induced by hepatic IRI is critical to develop novel therapeutics to attenuate acute-phase graft injury and prevent disease recurrence after liver transplantation. Targeting acute inflammation will not only rescue the early-phase graft dysfunction but also alleviate or prevent long-term complications. Therefore, it will be more efficient to kill two birds with one stone for the improvement in clinical outcome of patients after liver transplantation.

## 2. Acute-Phase Inflammation Initiated by Hepatic IRI

Liver IRI is the consequence of the temporary occlusion of blood supply followed by a sudden restoration of blood. Liver IRI includes two distinct stages, “ischemia” and “reperfusion”. Ischemia injury is a localized procedure of metabolic disturbance. In addition to metabolic disturbance, reperfusion injury results from profound inflammatory immune activations [5]. Cell death is the initial event which directly damages the cells during hepatic IRI. The release of DAMPs due to cell death changes the metabolism of hepatocytes and immune cells. The different cell death programs, alteration of mitochondrial metabolism and subsequent immune activation during hepatic IRI will be focused on. 

### 2.1. Cell Death

#### 2.1.1. Apoptosis and Necrosis

Apoptosis and necrosis are the two main types of liver cell death following hepatic IRI. The stimuli activate various proteins involved in apoptosis, such as caspase-3, caspase-8 and mitochondria cytochrome-C. These substances induce the destruction of DNA, finally resulting in apoptosis [6]. Previous studies reported that 50–70% endothelial cells and 40–60% hepatocytes appeared to undergo apoptosis during reperfusion [7,8]. However, some researchers argued that the predominant cell death was massive necrosis, particularly in steatotic livers. Some indicators suggested that both apoptosis and necrosis occurred simultaneously and even imbricated during hepatic IRI [9]. Our previous study indicated that aldose reductase (AR), a rate-limiting enzyme which can catalyze the NADPH-dependent reduction of glucose to sorbitol, increased the apoptosis in hepatic IRI [10]. One recent study found that tissue inhibitor of metalloproteinase 3 (TIMP3) deficiency induced cell death in hepatic IRI through the disruption of the hepatocyte E-cadherin/β-catenin complex [11]. New developments in exploring the mechanism of apoptosis/necrosis provide novel potential strategies against hepatic IRI. The regulation of Drp1 SUMOylation by augmenters of liver regeneration protected mitochondria from fission, rescuing hepatocytes from IRI-induced apoptosis [12]. Activating tripartite motif-containing 27 (TRIM27) prevented hepatocyte inflammation and apoptosis in hepatic IRI [13]. Hepatocyte-derived mesencephalic astrocyte-derived neurotrophic factor (MANF), an endoplasmic reticulum (ER) stress-inducible protein, alleviated hepatic IRI via regulating ER stress-induced apoptosis in mice [14]. Anti-complement 5 antibody (C5a) decreased the subsequent cascade not only by reducing platelet aggregation during the early phase but also by attenuating the activation of infiltrating macrophages/neutrophils and hepatocyte apoptosis in the late phase of reperfusion [15]. The mechanisms which connect apoptosis/necrosis and metabolism should be more noted in hepatic IRI.

#### 2.1.2. Ferroptosis

Ferroptosis is an iron-dependent form of cell death characterized by accumulation of intracellular lipid peroxide and imbalance of redox [16]. Ferroptosis has been reported as a contributor to the pathogenesis of hepatic IRI. From a retrospective clinical analysis of 202 pediatric living donor liver transplantations, a high serum ferritin level (a marker of iron overload) of the donor was found as an independent risk factor for hepatic damage post liver transplantation. Further investigation in the murine model indicated that upregulation of the ferroptosis marker prostaglandin endoperoxide synthase 2 (Ptgs2), liver damage and lipid peroxidation were induced by IRI and markedly reduced by the ferroptosis-specific inhibitors ferrostatin-1 or α-tocopherol. Moreover, hepatic IRI was alleviated by iron chelation via deferoxamine and exacerbated by iron overload through a high iron diet [17]. A recent study found that macrophage extracellular traps and ferroptosis were increased both in the patients that underwent hepatectomy with hepatic portal occlusion and in the mice subjected to hepatic IRI. Macrophage extracellular traps could aggravate ferroptosis [18]. Using liproxstatin-1, an inhibitor of ferroptosis, mitigated the damage induced by hepatic IRI [19]. These findings suggest the link of ferroptosis with immune responses during hepatic IRI.

#### 2.1.3. Pytoptosis

**Figure 1:**
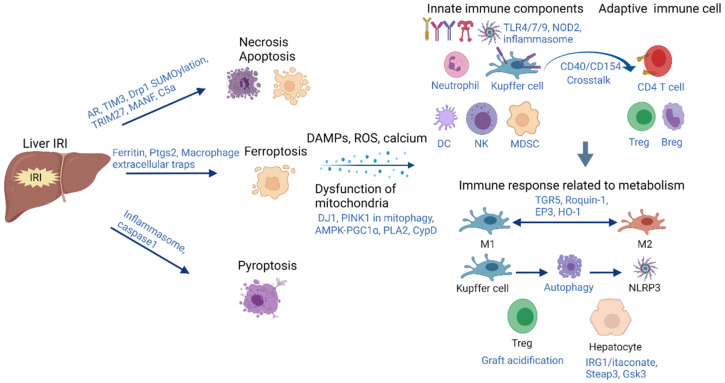
The inflammation induced by immune activation associated with metabolism dysfunction initiated by hepatic IRI during acute phase. Hepatic IRI leads to cell death, including necrosis, apoptosis, ferroptosis and pyroptosis. The dysfunction of mitochondria and the release of DAMPs, ROS and calcium initiates the immune responses. The PRRs (TLR4/7/9, NOD2, inflammasome) and immune cells (neutrophils, Kupffer cells, DC, NK, MDSC) contribute to the innate immune responses, which have crosstalk with adaptive immune activation (CD4^+^ T cell, Tregs, Bregs). The responses related to metabolism in immune cells contain the switch of M1–M2, autophagy and inflammasome activation in Kupffer cells, accumulation of Tregs, etc. In addition, the metabolism also plays critical roles in the immune responses of hepatocytes during the acute phase of hepatic IRI. IRI: ischemia reperfusion injury; AR: aldose reductase; TIMP3: metalloproteinase 3; TRIM27: tripartite motif-containing 27; MANF: mesencephalic astrocyte-derived neurotrophic factor; C5a: complement 5 antibody; Ptgs2: prostaglandin endoperoxide synthase 2; DAPMs: danger-associated molecular patterns; ROS: reactive oxygen species; PLA2: phospholipase A2; CypD: cyclophilin D; PRR: pattern recognition receptor; TLR: Toll-like receptor; NOD: nucleotide-binding oligomerization domain; DC: dendritic cell; NK: natural killer; MDSCs: myeloid-derived suppressor cells; M1: pro-inflammatory macrophage; M2: anti-inflammatory macrophage; TGR5: G protein-coupled bile acid receptor; HO-1: heme oxygenase 1; IRG1: immune-responsive gene 1; Steap3: six-transmembrane epithelial antigen of the prostate 3; Gsk3: glycogen synthase kinase 3.

Pyroptosis, distinguished from other forms of cell death, is a lytic type of cell death mediated by inflammasomes and caspase 1. In pyroptosis, cells become swollen, and activated caspase-1 cleaves full-length gasdermin *D* (GSDMD) to the GSDMD-N terminus, which oligomerizes and assembles into pores on the plasma membrane, leading to the release of a large amount of cell contents and initiating inflammatory responses. In both normal and steatotic liver, activated caspase-1 induces pyroptosis. Caspase 1/caspase 11 double-knockout attenuated liver IRI [20,21]. In addition to the hepatic injury during liver IRI, serum-derived exosomes from injured liver not only crossed the blood–brain barrier but also had similar effects on neuronal pyroptosis [22]. Thus, hepatic IRI could result in the pyroptosis of both the liver and neuronal system. Our previous study reported that the increase in nucleotide-binding oligomerization domain-like receptor family pyrin domain-containing 3 (NLRP3) inflammasomes induced liver IRI through activation of the telomere-independent repressor activator protein 1 (RAP1)/keratinocyte chemoattractant (KC) axis in neutrophils [23]. Inflammasome inhibitors (MCC950) could alleviate pyroptosis and injury [22]. Further mechanisms should be investigated to find novel targets against pyroptosis to mitigate hepatic IRI (Figure 1).

### 2.2. Dysfunction of Mitochondria and Metabolism Alteration during Hepatic IRI

Mitochondria constitute ~28% of the hepatocyte cell volume [24]. The dysfunctional mitochondria produce excessive reactive oxygen species (ROS), which in turn causes DNA damage, protein oxidation and lipid peroxidation, and ultimately results in cell death. During hepatic IRI, the balance between ROS and antioxidants shifts towards the former, resulting in oxidative stress and cytotoxicity [25]. In the initial phase (within 2 h after reperfusion), ROS appear to directly lead to hepatocellular injury. At the late phase (from 6 to 48 h post reperfusion), the induction of NADPH oxidase by neutrophils and Kupffer cells causes the production and release of ROS, which damages the hepatocytes [26]. The mitophagy, permeability change of mitochondria during hepatic IRI and metabolic alteration in IRI of steatotic liver will be noted.

#### 2.2.1. Mitophagy

Mitophagy is a form of selective autophagy for eliminating damaged mitochondria and is essential in maintaining mitochondrial homeostasis. DJ1 in hepatocytes but not myeloid cells decreased the protein stability of PARKIN, which reduced the onset of mitophagy resulting in the damaged mitochondria increase in liver IRI. DJ1 deficiency in hepatocytes could significantly ameliorate hepatic damage and inflammatory responses in IRI [27]. Mesenchymal stem cells (MSCs) ameliorated hepatocellular apoptosis mediated by PINK1-dependent mitophagy in hepatic IRI through adenosine monophosphate-activated protein kinase (AMPK)α activation [28]. This study was echoed by our recent finding, which reported that a compromised AMPK–PGC1α axis exacerbated steatotic graft injury by dysregulating mitochondrial homeostasis through impairment of biogenesis [29].

#### 2.2.2. Permeability Alteration of Mitochondria

The hypoxia in hepatic IRI impairs mitochondrial respiration and ATP synthesis, leading to calcium release from the ER into the cytosol [30]. The accumulation of cytosolic calcium causes increased calcium uptake into mitochondria and mitochondrial calcium overload, which results in mitochondrial permeability transition pore (MPTP) opening, depolarization and the initiation of cell death [31,32]. Inhibition of mitochondrial calcium uptake with ruthenium red significantly reduced liver injury in a rat model of IRI [33]. Preventing calcium release using the ryanodine receptor antagonist dantrolene improved the morphological maintenance of hepatic endothelial cells, the critical target of IRI [34]. In addition, antioxidants and prevention of calcium-induced MPTP formation or opening by edavarone or cyclosporine A were also found to decrease liver injury by indirectly regulating calcium overload, despite a lack of progress in clinical transplantation [35]. 

Hepatic IRI is associated with mitochondrial dysfunction and a reduction in phospholipids. Cytosolic phospholipase A2 (PLA2), a phospholipase, hydrolyzed mitochondrial membrane phospholipids and resulted in mitochondria-mediated oxidative stress and apoptosis. Ursodeoxycholyl lysophosphatidylethanolamide (UDCA-LPE) could regulate the crosstalk between the phospholipid metabolism and mitochondria, restore mitochondrial function and alleviate IRI [36]. During liver IRI, mitochondrial permeability transition pore opening leads to the dysfunction of mitochondria. Cyclophilin D (CypD), an isomerase that regulates the opening, could restore hepatic calcium retention capacity and oxidative phosphorylation parameters, reducing liver damage [37]. Zhou et al. found that high mitochondrial permeability led to high autophagy and exaggerated liver IRI, which indicated the cross talk of mitochondria and autophagy [38] (Figure 1).

#### 2.2.3. Metabolic Alteration in IRI of Steatotic Liver

The high incidence of non-alcoholic fatty liver disease (NAFLD) results in the frequent application of steatotic graft for liver transplantation due to the extreme shortage of donors [39]. The quantification and grading of liver steatosis are classified as mild, moderate or severe if less than 30%, 30–60% or more than 60%, respectively, of hepatocytes display fat infiltration [40,41]. The previous findings indicate that steatotic livers with less than 60% fatty infiltration could be accepted for liver transplantation. The mildly (<30%) steatotic grafts should be used routinely as most livers with mild steatosis (<30%) neither affected long-term graft function nor patient survival. Caution should be applied using organs with moderate (30–60%) steatosis because primary graft dysfunction was more prevalent despite the controversial effects on survival [42].

Although the effects of steatosis on liver transplantation might be controversial, more evidence indicated that steatosis might worsen hepatic IRI. Increased macrovesicular steatosis (>30%) was associated with increased histological damage, hepatic function derangement and reduced survival after liver transplantation according the systemic review [43]. Moreover, our recent study demonstrated that graft steatosis over 10% was an independent risk factor for poor post-transplant survival and was associated with acute graft injury after living donor liver transplantation (LDLT) [29]. Along with the mechanism underlying NAFLD being clarified, the mechanisms of severe IRI in steatotic grafts are paid more attention [44]. The significant roles of Wnt4 and Lipocalin2 signaling were illustrated in small-for-size fatty graft injury [45,46]. In terms of cellular mechanisms, AR and RAP1 promoted small-for-size fatty graft injury via inducing intrahepatic macrophages activation and recruiting neutrophils, respectively [10,47]. High levels of RAP1/KC/NLRP3 inflammasome in neutrophils contributed to the small-for-size fatty graft injury resulting in poorer liver function post-transplantation [23]. Our another study found that IL-17a exacerbated hepatic IRI in fatty liver by promoting neutrophil infiltration and mitochondria-driven apoptosis [48]. Hepatic hyperlipidosis causes elevated cytosolic and reduced endoplasmic calcium levels, which results in ER stress and unfolded protein response in the development and progression of NAFLD [35]. However, the role of calcium signaling in steatotic liver IRI has not been clarified and should be noted in the future study.

### 2.3. Immune Responses Associated with Metabolic Alteration during Hepatic IRI

During hepatic IRI, transient cell death and dysfunction of mitochondria induces the production of DAMPs. These DAMPs initiate the innate immune responses which are predominant in the inflammation induced by IRI. Recently, the pattern recognition receptors (PRRs) induced by liver IRI were screened in clinical biopsies, and Toll-like receptor (TLR) 4/7/9 and nucleotide-binding oligomerization domain (NOD)2 were found to be involved in either promoting or attenuating hepatic IRI [49]. Consistently, it was reported that intestinal TLR9 deficiency resulted in exacerbated hepatic IRI with increased small intestinal apoptosis and inflammation [50]. In addition to innate immune activation, CD4^+^ T cells are also involved in the inflammation initiated by hepatic IRI, which demonstrates the key role of adaptive immune responses [4]. The crosstalk between innate and adaptive immune responses leads to hepatic IRI [5]. This complex process involves multiple immune cells such as neutrophils, Kupffer cells, dendritic cells (DC), natural killer cells (NK cells), myeloid-derived suppressor cells (MDSCs), CD4^+^ T cells, Tregs and Bregs, etc. The metabolism and immune responses have been noted in recent years. In this review, we mainly discuss the inflammation induced by immune responses related to metabolic alteration during hepatic IRI.

#### 2.3.1. Kupffer Cells and Tregs

Kupffer cells, macrophages in liver, play an important role in the pathogenesis of hepatic IRI. Metabolism in macrophages affects the polarization of M1/M2. It was reported that the depletion of plasma membrane-bound G protein-coupled bile acid receptor (TGR5) in myeloid cells aggravated liver injury with enhanced mobility and proinflammatory M1 polarization of macrophages. IR stress promoted TGR5 activation in CD11b^+^ cells, correlating with the shift in M2 polarization. Therefore, TGR5 attenuated proinflammatory immune activation by restraining macrophage migration and facilitating M2 polarization [51]. Roquin-1 increase induced by hepatic IRI promoted the polarization of M2 and inhibited the polarization of M1, which described the protection of liver IRI through Roquin-1 at the acute phase [52]. EP3, the prostaglandin E (PGE) receptor in monocyte-derived DCs, facilitated liver repair by inducing IL-13-mediated switching of the macrophage phenotype from pro-inflammatory to pro-reparative in hepatic IRI [53]. Moreover, myeloid heme oxygenase 1 (HO-1) expression regulated macrophage polarization, at least partially, through favoring an M2 phenotype, protecting against liver IRI [54]. In addition to polarization, a study indicated that enhancing the autophagy of Kupffer cells inhibited the activation of NLRP3 caused by IRI, and inhibiting autophagy induced the secretion of IL-1β dependent on NLRP3 activation [55]. CEACAM1 (CC1) glycoprotein functions at the interface of immune liver injury and metabolic homeostasis, and it was recently found that its low levels were associated with increased ROS and HMGB1 translocation, enhanced innate and adaptive immune responses and inferior early liver graft function in the liver biopsies of human donors. Hepatic flush from CC1-deficient livers enhanced macrophage activation and augmented cold-stress-triggered ASK1/p-p38 increase. CC1 might be a checkpoint regulator of IR stress and sterile inflammation [56]. A report indicated that hepatic IRI induced microenvironment acidification and dysfunction of the liver in humans and mice. The acidic microenvironment could inhibit the generation and function of CD4^+^CD25^+^Foxp3^+^ Tregs. The reversal of the acidic microenvironment restored Foxp3 expression and Treg function. Using omeprazole, a proton pump inhibitor, improved the decreased Treg differentiation induced by the acidic microenvironment during hepatic IRI [57]. The accumulation of anti-inflammatory immune cells such as Tregs and M2 macrophages during the acute phase may inhibit inflammation. However, these cells may change the immune regulation of the liver graft microenvironment in the long term, which favors liver cancer recurrence and fibrogenesis. Thus, the balance of immune cells should be considered for the long-term outcome.

#### 2.3.2. Hepatocytes

In addition to the immune cells, the immune responses associated with metabolism are also initiated in hepatocytes. Immune-responsive gene 1 (IRG1) encodes for the enzyme producing itaconate, which is a metabolite of the tricarboxylic acid cycle and plays an anti-inflammatory role in macrophages. However, Yi et al. recently found that the IRG1/itaconate pathway activated Nrf2-mediated antioxidative response in hepatocytes (nonimmune cells) to protect the liver from IRI. Deletion of IRG1 exacerbated liver injury and systemic inflammation [58]. Six-transmembrane epithelial antigen of the prostate 3 (Steap3), a key regulator of iron uptake, was reported as the mediator of hepatic IRI in hepatocytes by regulating inflammatory responses as well as apoptosis through TAK1-dependent activation of the JNK/p38 pathways [59]. Glycogen synthase kinase 3 (Gsk3) N-terminal serine phosphorylation inhibited liver innate immune activation and decreased hepatocyte autophagy in response to inflammation. Moreover, the mutation of Gsk3α, instead of Gsk3β, protected against IRI in liver parenchyma, but exacerbated hepatocellular damage in non-parenchymal cells, which indicated the isoform- and cell type-specific roles of Gsk3 in hepatic IRI [60] (Figure 1).

## 3. Long-Term Impact of Inflammation Induced by Liver IRI

Acute-phase inflammation is not only detrimental during the early stage post liver transplantation, but also alters the immune microenvironment in the liver graft, which leads to chronic inflammation. The long-term impacts include chronic graft damage, biliary complications, impairment of regeneration, progression to liver cancer recurrence and graft fibrosis. Here, we mainly discuss liver cancer recurrence and fibrogenesis. 

### 3.1. Cancer Recurrence

Tumor recurrence caused significantly poor outcome in hepatocellular carcinoma (HCC) patients that underwent liver transplantation [61]. In addition to the tumor biology, both clinical and animal studies have suggested that accelerated acute-phase inflammation due to hepatic IRI promoted tumor recurrence through upregulating cell signaling for tumor cell adhesion, invasion and angiogenesis [62,63]. A retrospective study of clinical analysis reported that prolonged cold (>10 h) and warm (>50 min) ischemia times were independent risk factors for HCC recurrence [63]. One systematic review with meta-analysis including 8087 patients indicated that the patients that received liver pedicle clamping had significantly shorter survival and higher tumor recurrence rates [64]. In the previous studies, we found that transient portal hypertension induced destruction of the hepatic sinusoidal endothelium and the initial shear stress resulted in inflammatory cascades in small-for-size liver grafts [65,66,67]. The grafts from living donors (often small-for-size to the recipient) or steatotic liver are more susceptible to IRI with exacerbated inflammation. The severe inflammation induced by IRI in marginal grafts not only leads to early- or late-phase graft dysfunction but also raises the risk for tumor recurrence in liver cancer patients [68,69].

The injured liver graft (soil) may provide a favorable environment for tumor recurrence. Simultaneously, the inflammatory signaling may activate the invasive properties of tumor cells (seeds) [70]. The hypoxia post liver surgery causes the rapid dedifferentiation of tumor cells into immature cancer stem cells accompanied with high clone- and metastasis-forming capacity, promoting invasion and accelerating metastatic outgrowth [71]. C-reactive protein (CRP), a biomarker of inflammation, was identified as an independent predictor of poor HCC recurrence-free survival. Thus, early postoperative serum CRP can serve as an inflammation-based biomarker of outcome for liver transplant patients with HCC [72]. CXCL10 was initially found to be required for the induction of pro-inflammatory responses in hepatic IRI [73]. In addition, CXCL10 could, through its receptor CXCR3, induce the mobilization, differentiation and angiogenesis of endothelial progenitor cell (EPC), which resulted in progressive tumor growth [74]. Moreover, Tregs could be also recruited to the liver graft by the inflammatory signal CXCL10/CXCR3 at the acute phase, which promoted the HCC recurrence. The knockout of CXCL10 and depletion of Tregs inhibited tumor recurrence post reperfusion [75]. Our recent study found that monocytic MDSCs were recruited by CXCL10 through TLR4, instead of CXCR3, to promote HCC recurrence. The recruitment of MDSC was dependent on the motility gene MMP14. CXCL10 or TLR4 deficiency could effectively reduce the liver tumor progression after IRI [76]. CD40 was induced by macrophages responding to TLR4 and type I interferon (IFN) stimulation during hepatic IRI. CD40 upregulation triggered the engagement of CD154–CD40. CD4^+^ T cells facilitated hepatic inflammation and injury through CD154 without de novo Ag-specific activation [77]. Regulatory B cells (Bregs) promoted HCC growth and invasiveness by directly interacting with liver tumor cells through the CD40/CD154 signaling pathway, which may suggest the role of Bregs in tumor recurrence [78]. M2 macrophages were reported to promote HCC invasiveness and recurrence in our previous study [79]. Balancing the inflammatory factors induced by hepatic IRI to switch between M1 and M2 macrophages was critical to prevent HCC recurrence. One study based on the effects on the gut–liver axis indicated that portal triad clamping provoked venous engorgement and enhanced bacterial translocation, resulting in aggravated tumor burden. This observation was associated with the LPS–TLR4 pathway [80]. 

**Figure 2:**
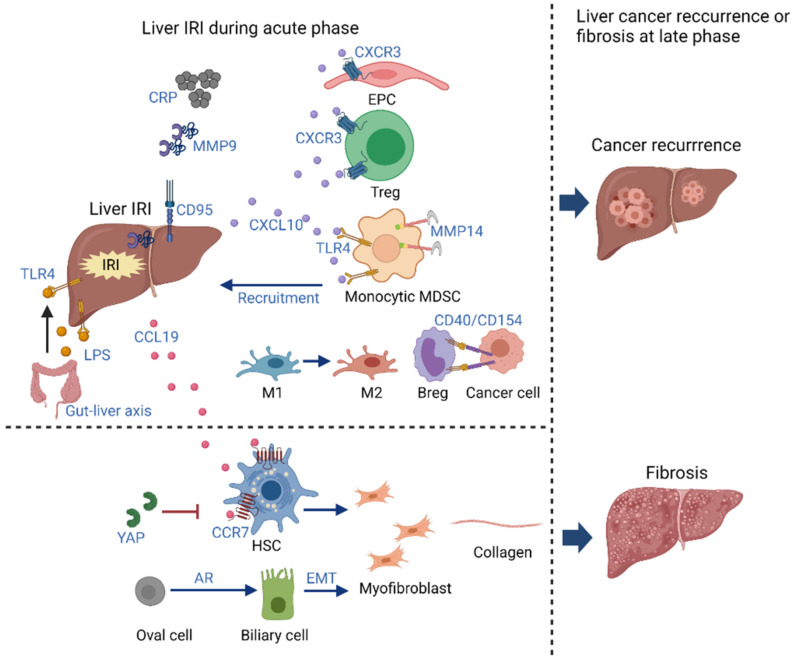
The long-term impact of inflammation induced by immune responses in hepatic IRI. The inflammation induced by hepatic IRI during acute phase results in liver tumor recurrence via CXCL10 recruiting EPCs, Tregs and MDSCs through CXCR3 or TLR4, M1 switching to M2, Bregs interacting with cancer cells, LPS stimulation through TLR4 in gut–liver axis, the deregulation of CRP, MMP9, CD95, etc. Early liver IRI leads to fibrogenesis at late phase through CCL19/CCR7 axis regulating HSCs, EMT of oval-cell-derived biliary cell by AR activation, myofibroblast accumulation and collagen formation, which can be inhibited by YAP. IRI: ischemia reperfusion injury; chemokine (C-X-C motif) ligand 10 (CXCL10); C-X-C motif chemokine receptor 3 (CXCR3); TLR: Toll-like receptor; EPC: endothelial progenitor cell; MDSCs: myeloid-derived suppressor cells; CRP: C-reactive protein; MMP: metalloproteinase; LPS: lipopolysaccharide; M1: pro-inflammatory macrophage; M2: anti-inflammatory macrophage; HSCs: hepatic stellate cells; CCL19: chemokine (C–C motif) ligand 19; CCR7: CC-chemokine receptor 7; AR: aldose reductase; EMT: epithelial-to-mesenchymal transition.

In addition to primary liver cancer, surgical resection remains the best treatment for colorectal metastases isolated to the liver. However, liver cancer recurrence was a significant reason for treatment failure. The IRI incurred during liver surgery leads to cellular dysfunction and elevation of proinflammatory cytokines, which may contribute to liver tumor recurrence. In the clinical analysis of colorectal liver metastases, ischemia grade 2 or higher of remnant liver was associated with worse cancer-specific survival after liver resection [81]. An early study used a highly standardized mouse model of partial hepatic IRI to study the effects of IRI on the colorectal micrometastases. They found that the outgrowth of micrometastases in occluded liver lobes was accelerated five- to six-fold, in contrast with nonoccluded lobes, and was associated with areas of necrotic liver surrounded by inflammatory cells and apoptotic hepatocytes. Furthermore, the accelerated tumor progression and tissue necrosis were completely prevented by occluding blood flow intermittently [82]. Mice subjected to 30 min of 70% liver ischemia at the time of tumor inoculation had significantly larger tumor number and volume with higher levels of MMP9 in the serum and liver tissue. The results indicated that hepatic IRI-induced MMP9 increase contributed to the growth of metastatic colorectal carcinoma in the liver [83]. Activation of the CD95 system not only contributes to liver IRI, but also promotes the accelerated outgrowth of colorectal liver metastases [84]. Prevention of postischemic microcirculatory disturbances and perinecrotic hypoxia decreased the colorectal liver metastases with the disturbance of HIF-1 alpha stabilization post IRI [85] (Figure 2).

### 3.2. Fibrosis

Fibrosis post liver transplantation is associated with impaired liver function and poor graft survival. In the mouse hepatic IRI model, the levels of profibrotic genes increased during the hepatic remodeling. α-smooth muscle actin (α-SMA)-positive hepatic stellate cells (HSCs)/myofibroblasts were more infiltrated and collagen deposition was enhanced along the injured site. HSCs, instead of portal fibroblasts, were identified as the major source of myofibroblasts. Liver fibrosis was observed at the interface between necrotic tissue and regenerating liver [86]. In our recent study, IRI and fibrosis were both enhanced in cytomegalovirus latency of patients and a rat model after transplantation. Mechanistically, the increase in CCL19/CCR7 not only induced IRI, but also promoted the migration of hepatic stellate cells to aggravate liver fibrogenesis [87]. In addition to the role in acute hepatic injury, AR activated the oval cells, which could differentiate into biliary cells undergoing epithelial-to-mesenchymal transition to promote liver fibrogenesis [88]. IRI induced higher expressions of Yap, which protected the liver against IRI. Moreover, YAP activation inhibited extracellular matrix synthesis and diminished HSC activation, resulting in the prevention of fibrosis. These results document the coincident role of YAP in IRI and subsequent liver fibrogenesis [89] (Figure 2).

## 4. Therapeutic Strategies to Reduce Both Short- and Long-Term Impacts of Inflammation in Hepatic IRI

The therapeutic strategies against liver IRI mainly include surgery, pharmacological strategies and perfusion machine. As one of the surgical strategies, ischemic preconditioning is a promising clinical application [90,91,92]. Among various kinds of pharmacological strategies (antioxidants, adenosine agonists, pentoxifylline, protease inhibitors, prostaglandins, MMPs inhibitors and FTY720, etc.) based on the studies in animal models, pentoxifylline could be a potential agent as it has been used for many years in the treatment of peripheral disease [92,93,94,95,96,97,98,99]. Furthermore, some pharmacological strategies (epoprostenol, thymoglobulin, HEGPOL and rPSGL-Ig, etc.) have been applied in randomized clinical trials [100,101,102,103]. In addition to the progress in animal models, a normothermic perfusion machine was associated with a 50% lower level of graft injury in clinical trials, providing great potential application in clinical practice [104,105]. As mentioned above, the inflammation initiated by hepatic IRI not only induces acute-phase hepatic injury but also results in cancer recurrence and fibrogenesis in the long term. Thus, strategies which can decrease both the short- and long-term impacts of inflammation induced by hepatic IRI are efficient and promising. Ischemia preconditioning, pharmacological inhibitors and machine perfusion, which can decrease both the short- and long-term impacts of inflammation induced by hepatic IRI, will be discussed here. 

### 4.1. Ischemic Preconditioning

Ischemic preconditioning has been applied to ameliorate hepatic IRI in clinical studies [90]. Strong evidence certified that adenosine was a critical mediator in ischemic preconditioning. Adenosine activated the adenosine A2 receptor, which initiated nitric oxide (NO) [93,106,107,108]. NO induced the activation of protein kinase C, AMPK and p38 MAPK [109,110,111,112]. Activation of these signaling pathways not only increased the tolerance of hepatocytes and endothelial cells against ischemic insults but also led quiescent cells to enter the cell cycle and initiated the regenerative responses [112]. Moreover, in steatotic liver, ischemic preconditioning before IRI decreased the tumor burden to the level of that in non-ischemic controls. This protective effect was associated with inhibited cancer cell motility [68]. Remote ischemic preconditioning protected the intestinal integrity and decreased bacterial translocation, thereby mitigating HCC recurrence [80]. During liver transplantation, the pre-retrieval reperfusion of ischemia donors partially reversed the IRI lesions in contrast with non-reperfusion ischemic donors in the rat model. Simultaneously, HCC growth was higher in the non-reperfusion group and was prevented in the pre-retrieval reperfusion group. Furthermore, in the ischemic-only donor group, there was a stronger inflammatory cytokine profile, higher hypoxia and HCC growth-enhancer genes, including Hmox1, Hif1a and Serpine1 [113]. The surgical strategies against hepatic IRI, especially preventing the long-term effects, should be further investigated.

### 4.2. Pharmacological Inhibitors

Prostaglandin E (PGE)1 treatment has been shown to decrease hepatic IRI in liver transplant patients. Targeting microsomal PGE1 could facilitate liver repair and polarize macrophages into the anti-inflammatory type after acute liver injury [114]. A retrospective review of 106 liver transplant patients with HCC indicated that 3- and 5-year recurrence-free survival rates were significantly higher in the PGE1 group in contrast to the non-PGE1 population. Moreover, PGE1 treatment was an independent promoter of recurrence-free survival [115]. An interesting study using antibiotics pretreatment decreased ER stress, enhanced autophagy and inhibited inflammation, whereas it increased serum PGE2 and hepatic PGE2 receptor 4 (EP4) expression. These results document the benefits of antibiotic pretreatment in liver transplant recipients and point to the microbiome as a therapeutic target [116]. Studies from gut-sterilized and germ-free mice, as well as mice treated with microbial metabolites or microbiota-associated molecular patterns, have certified that the microbiota and microbially activated pathways contributed to the development of HCC, despite no clinical evidence [117]. Thus, antibiotic administration may decrease hepatic IRI and HCC recurrence after transplantation. TLR4 initiates the inflammatory cascades during hepatic IRI. TLR4 inhibition could effectively alleviate inflammation during the acute phase of liver IRI [118]. Our recent study demonstrated that TLR4 inhibition could significantly decrease tumor progression with decreased monocytic MDSCs in the liver tumor recurrence model post IRI [76]. These findings indicate that TLR4 might be a potential target. 

In addition to the pharmacological inhibitors, MSCs and their derivatives can also be used to inhibit the short- and long-term effects initiated by hepatic IRI. Adoptive transfer of MSCs increased CD47 expression and mitigated liver IRI. The depletion of CD47 in MSCs exacerbated IRI-induced liver damage [119]. Extracellular vesicles derived from human umbilical cord MSCs protected against hepatic IRI by suppressing oxidative stress and neutrophil inflammatory response. Mechanistically, manganese superoxide dismutase (MnSOD), the mitochondria-located antioxidant enzyme, was encapsulated in the treatment of extracellular vesicles and decreased oxidative stress [120]. Exosomes derived from human umbilical cord MSCs reduced the number of surface fibrous capsules and caused their texture to become soft, mitigating the inflammation and collagen deposition in fibrotic liver. Exosomes also significantly ameliorated liver function, and reduced collagen type I and III, transforming growth factor (TGF)-β1 and phosphorylation Smad2 level. Moreover, EMT-associated markers, and subsequently fibrosis in the liver, were inhibited by the treatment of exosomes [121]. In our previous study, using MSCs derived from human-induced pluripotent stem cells (hiPSC) as a vehicle, glutathione peroxidase 3 (Gpx3) could significantly decrease hepatic IRI by inhibition of hepatocyte apoptosis and senescence. Furthermore, we identified that the decrease in GPx3 in small-for-size grafts was associated with tumor progression both in clinical and animal studies. Real-time intravital imaging demonstrated that GPx3 effectively inhibited HCC invasiveness in a live animal [122]. Thus, GPx3 is a potential target due to its beneficial role against hepatic IRI and subsequent HCC recurrence.

### 4.3. Machine Perfusion

Machine perfusion has been recognized as one of the significant improvements in liver transplantation. There are two main perfusion types. One type is perfusion with blood or alternative oxygen carriers at physiologic, normothermic or sub-normothermic conditions. The other is perfusion with cooled, oxygenated artificial fluids [123]. Hypothermic oxygenated perfusion (HOPE) could reduce liver IRI by decreasing hepatocyte injury, Kupffer cell and endothelial cell activation. Moreover, patients receiving donations after cardiac arrest treated with HOPE were protected from biliary injury [124]. For allogeneic liver transplantation, HOPE could not only protect against preservation injury but also decreased the alloimmune responses in a rat model [125]. Regarding the marginal graft, HOPE treatment markedly ameliorated IRI with decreased oxidative stress, less nuclear injury, reduced Kupffer and endothelial cell activation and less fibrosis within one week post transplantation [126]. In addition to the studies in animal models, a recent clinical retrospective study demonstrated that low total hepatic flow was significantly associated with tumor recurrence, indicating that graft hemodynamics affect HCC recurrence after liver transplantation [127]. Due to the mechanistic link between IRI and HCC recurrence, dynamic liver preservation approaches may serve as promising strategies to decrease HCC recurrence. A clinical analysis indicated that the tumor recurrence rate was four-fold higher in unperfused donation after brain death (DBD) livers (25.7%), compared to 5.7% of recipients with tumor recurrence in the HOPE-treated donation after circulatory death (DCD) controls. The 5-year tumor-free survival in HCC recipients of DCD livers was 92% with HOPE treatment, compared to 73%, 82.7% and 81.2% in patients with unperfused DBD or DCD livers. These results suggest that machine perfusion might be promising due to its therapeutic effect on both the short- and long-term impact of hepatic IRI [128]. 

## 5. Conclusions and Perspectives

During hepatic IRI, the release of DAMPs due to cell death, mitochondrial dysfunction and disturbance of metabolic homeostasis initiates the immune responses and inflammation. The inflammation not only causes acute hepatic damage but also leads to long-term complications, such as HCC recurrence and fibrogenesis post liver transplantation. Therapeutic strategies including ischemic preconditioning, pharmacological inhibitors and machine perfusion against both acute inflammatory injury and long-term detrimental impact should be studied further to efficiently improve outcomes for liver transplant patients.
